# Signet-ring cell carcinoma of the appendix presenting as diffuse uterine involvement: a case report and literature review

**DOI:** 10.3389/fonc.2026.1833728

**Published:** 2026-04-20

**Authors:** Kaige Pei, Junhan Liu, Chen Ling

**Affiliations:** 1 Department of Gynecology and Obstetrics, West China Second University Hospital, Sichuan University, Chengdu, Sichuan, China; 2 Key Laboratory of Obstetrics and Gynecologic and Pediatric Diseases and Birth Defects of Ministry of Education, West China Second University Hospital, Sichuan University, Chengdu, Sichuan, China

**Keywords:** appendiceal neoplasms, cytoreductive surgery, pseudomyxoma peritonei, signet-ring cell carcinoma, uterine metastasis

## Abstract

**Background:**

Appendiceal signet-ring cell carcinoma (SRCC) is rarely encountered, and presentation with diffuse uterine involvement as the initial manifestation has not been systematically reported, posing significant risk of misdiagnosis as primary uterine malignancy.

**Case presentation:**

A 45-year-old woman presented with vaginal bleeding for 4 months. Preoperative CT demonstrated diffuse uterine myometrial thickening and appendiceal wall thickening. Curettage pathology revealed signet-ring cells with immunohistochemistry CK20+++/SATB-2+++/CK7-/PAX-8-, favoring gastrointestinal origin. Intraoperatively, the uterus was 16-week size with a firm, enlarged appendix and right ovarian involvement. Cytoreductive surgery (CRS) confirmed appendiceal SRCC with uterine and mesenteric metastases. Eight cycles of adjuvant 5-FU plus cisplatin were administered. First recurrence occurred at 32 months, treated with six cycles of 5-FU, carboplatin, and bevacizumab. At last follow-up (January 2026), overall survival exceeded 52 months with normalized tumor markers.

**Conclusions:**

For patients with “uterine enlargement and ascites,” appendiceal SRCC should be suspected. The enteric immunophenotype and right-sided ovarian dissemination pattern are diagnostic keys. Thorough surgery combined with chemotherapy can achieve long-term survival.

## 1 Introduction

Appendiceal signet-ring cell carcinoma (SRCC) represents an extremely rare and highly aggressive subtype of gastrointestinal malignancy, accounting for less than 0.1% of all gastrointestinal tumors (1) and approximately 4% of primary appendiceal carcinomas (2, 3). Its clinical diagnosis poses substantial challenges, primarily because symptoms overlap considerably with those of acute appendicitis, lacking specificity (4, 5). Patients typically present with right lower quadrant pain, and imaging studies often demonstrate nonspecific findings such as appendiceal wall thickening and surrounding inflammation, resulting in extremely low preoperative diagnostic rates. Most cases are discovered incidentally through postoperative pathological examination following appendectomy (5, 6). This diagnostic delay frequently means disease has progressed to an advanced stage, with approximately 61.3% to 69% of patients having metastases at initial diagnosis (7–9), thereby significantly compromising treatment efficacy and patient prognosis.

Epidemiologically, primary appendiceal adenocarcinoma represents a rare gastrointestinal malignancy with an estimated incidence of 0.08–0.12 cases per 100,000 population annually, accounting for approximately 0.5–1.0% of all gastrointestinal neoplasms and 0.9–1.4% of appendiceal pathologies (10). The WHO classification recognizes four main histologic subtypes: mucinous adenocarcinoma (approximately 40–50% of cases), non-mucinous (intestinal-type) adenocarcinoma (30–35%), signet-ring cell carcinoma (4–5%), and mixed types. Mucinous adenocarcinomas frequently arise from precursor lesions—low-grade appendiceal mucinous neoplasms (LAMN) or high-grade appendiceal mucinous neoplasms (HAMN)—through a well-characterized adenoma-carcinoma sequence driven by distinct molecular alterations (11).

Pathogenetically, appendiceal mucinous neoplasms demonstrate a unique molecular signature characterized by high-frequency KRAS mutations (present in 70–90% of LAMN and 40–60% of mucinous adenocarcinomas) and GNAS activating mutations (identified in 20–50% of cases) (12, 13). These mutations synergistically promote mucin hypersecretion and neoplastic progression through constitutive activation of the MAPK/ERK and cAMP/PKA signaling pathways, respectively (14). The accumulation of intraluminal mucin leads to appendiceal distension, potential perforation, and subsequent peritoneal dissemination—collectively termed pseudomyxoma peritonei (PMP) (15). In contrast, signet-ring cell carcinoma typically exhibits more aggressive biological behavior, frequently demonstrating TP53 mutations and chromosomal instability rather than the KRAS/GNAS-mutant profile of well-differentiated mucinous tumors (16). This molecular dichotomy underlies the substantially different clinical outcomes: while well-differentiated mucinous adenocarcinoma with PMP may achieve 10-year survival rates exceeding 70% following complete cytoreduction, SRCC portends a dismal prognosis with median survival historically reported as 12–24 months (7, 17).

The biological behavior of appendiceal SRCC is highly aggressive, with early peritoneal surface dissemination leading to pseudomyxoma peritonei (PMP), clinically manifesting as ascites, peritoneal thickening, and pelvic organ involvement, while the primary site remains occult and easily misdiagnosed. The uterus, as one of the pelvic organs most frequently affected by PMP, demonstrates two fundamentally distinct patterns of involvement with markedly different clinical implications: (1) Primary uterine SRCC is exceedingly rare, with only sporadic cases reported globally, arising from variants of gastric-type endocervical adenocarcinoma or endometrial mucinous carcinoma with signet-ring cell features, carrying a dismal prognosis but requiring relatively limited surgical resection (18); (2) Metastatic SRCC involving the uterus typically originates from the appendix or colorectum, representing advanced peritoneal dissemination requiring radical cytoreductive surgery (CRS), with completely different treatment strategies and prognostic expectations (17). However, these two entities overlap substantially in clinical presentation (vaginal bleeding, uterine enlargement), imaging findings (diffuse uterine myometrial thickening, ascites), and histomorphology (signet-ring cells, mucin pools), rendering preoperative differentiation extremely challenging. Misclassification risks inadequate surgical resection (treating metastasis as primary) or overtreatment (treating primary as metastasis), directly compromising patient survival.

Therefore, for SRCC presenting with diffuse uterine involvement as the initial manifestation, systematic preoperative evaluation for appendiceal and colorectal primaries, intraoperative frozen-section immunophenotyping, and postoperative pathological tracing of the invasive gradient are essential. This case, ultimately diagnosed as appendiceal SRCC with uterine metastasis through integrated imaging, surgical, pathological, and immunohistochemical evidence, aims to inform the diagnosis and management of similar cases.

## 2 Case presentation

### 2.1 Patient information

A 45-year-old woman, gravida 2 para 1, presented with “irregular vaginal bleeding for more than 4 months, uterine lesion discovered 25 days ago” and was admitted on July 5, 2021. Four months prior, she had undergone fractional curettage at an outside hospital for irregular vaginal bleeding, with pathology showing “endometrial simple hyperplasia, focal polyp formation, chronic cervicitis,” and vaginal bleeding persisted postoperatively. Twenty-five days before admission, bleeding increased with clots. Her grandfather had died of gastric cancer.

### 2.2 Clinical findings

Physical examination revealed stable vital signs and a Pfannenstiel scar in the lower abdomen. Gynecologic examination demonstrated normal external genitalia; patent vagina with scant bleeding; enlarged, smooth, firm cervix; anteverted uterus, 16-week gestation size, firm, with limited mobility and mild tenderness; thickened, tender left adnexa; unremarkable right adnexa. Cervical cytology showed atrophic inflammatory changes.

### 2.3 Timeline

See **Table 1**.

**Table 1:** Clinical timeline of a 45-year-old patient with appendiceal signet-ring cell carcinoma presenting as diffuse uterine involvement.

Date	Event
Apr 23, 2021	Fractional curettage at outside hospital: endometrial hyperplasia
Jun 11, 2021	Contrast-enhanced CT: thickened uterine myometrium (7.9×7.5×8.9 cm); thickened, enhancing appendix (1.0 cm); minimal pelvic effusion
Jun 15, 2021	Pathology review: signet-ring cells in cervix and endometrium; IHC CK20+++, CK7–, PAX-8–; favor metastatic carcinoma of gastrointestinal origin
Jun 28, 2021	Esophagogastroduodenoscopy: chronic non-atrophic gastritis; Colonoscopy: no abnormalities
Jul 5, 2021	Admission to our hospital
Jul 6, 2021	Cytoreductive surgery (see 2.5)
Jul 26, 2021	Final pathology: appendiceal signet-ring cell carcinoma with uterine, mesenteric, and omental metastases; AJCC Stage IVC (pT4aNxM1), PCI 16, CC-0
Aug 2021–Apr 2022	Adjuvant chemotherapy: 8 cycles of 5-FU + cisplatin (intraperitoneal)
Feb 2022	PET/CT: no residual hypermetabolic foci; omental stranding improved
Mar 2024	First recurrence: PET/CT showed nodular thickening at vaginal cuff and right sacral ligament with increased FDG uptake
Mar–Sep 2024	Second-line chemotherapy: 6 cycles of 5-FU + carboplatin + bevacizumab
Jan 2026	Last follow-up: asymptomatic; tumor markers normal (CEA 0.2 ng/mL, CA19-9 18.7 U/mL, CA-125 17.8 U/mL)

### 2.4 Diagnostic assessment

**Figure 1:**
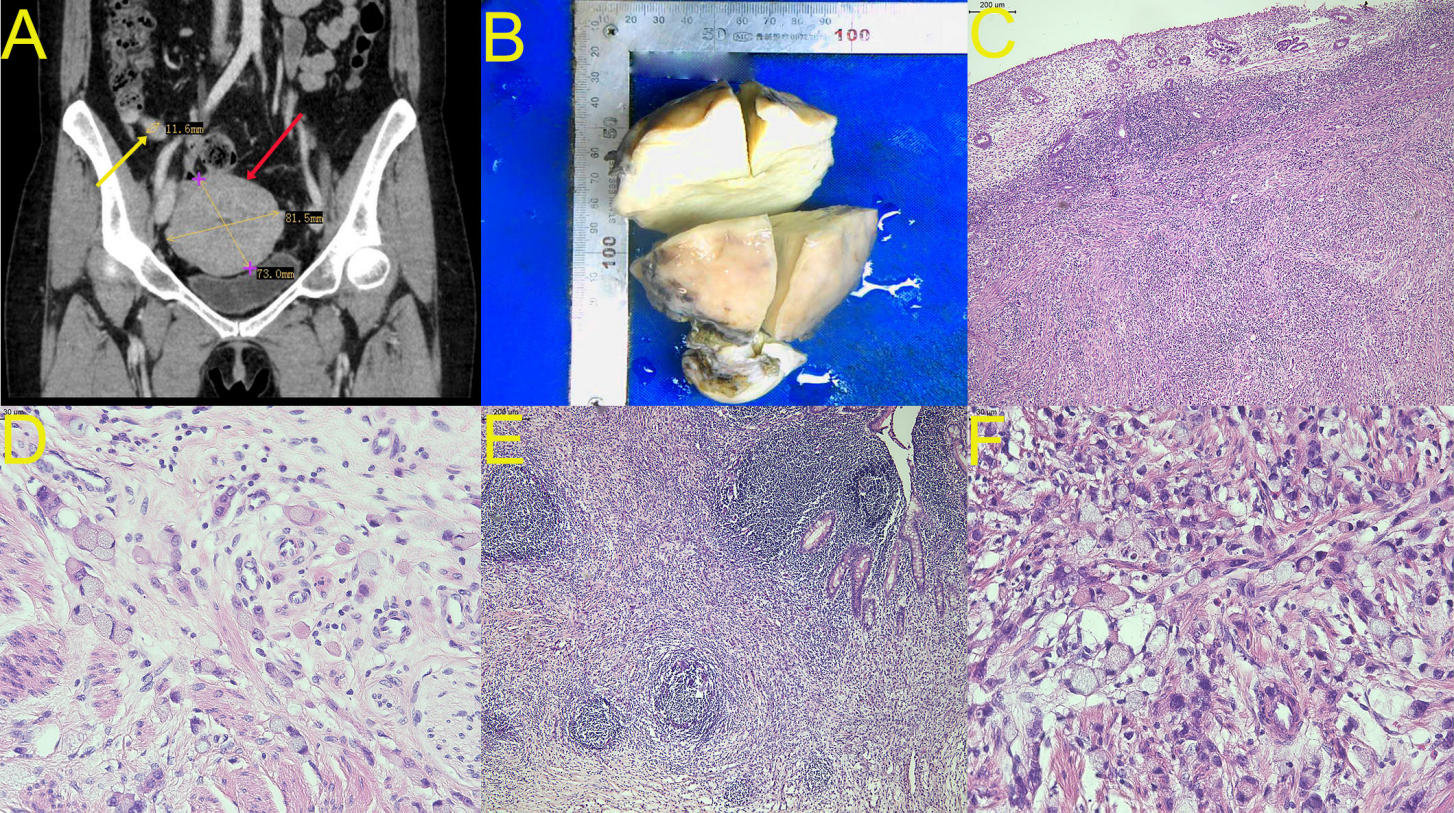
Gross, radiological and histopathological features. **(A)** Preoperative contrast-enhanced CT (coronal view) demonstrating markedly enlarged uterus (red arrow, 81.5 × 73.0 mm) and thickened appendix (yellow arrow, 11.6 mm; normal <6 mm)—the critical “imaging blind spot” that was initially overshadowed by prominent uterine findings. **(B)** Gross specimen of the uterus showing enlarged, firm uterus with solid, yellow-white cut surface. **(C)** Hematoxylin and eosin (H&E) staining of uterine myometrium at 40× magnification showing the diffuse architectural pattern of infiltration by signet-ring cells. **(D)** H&E staining of uterine myometrium at 200× magnification demonstrating at higher power the classic signet-ring cells with eccentric nuclei and abundant intracytoplasmic mucin. **(E)** H&E staining of appendix at 40× magnification showing transmural infiltration by signet-ring cell carcinoma. **(F)** H&E staining of appendix at 200× magnification revealing abundant signet-ring cells with mucin vacuoles displacing nuclei to the periphery.

Preoperative contrast-enhanced CT (June 11, 2021) demonstrated diffuse uterine myometrial thickening with heterogeneous enhancement, appendiceal wall thickening with enhancement (transverse diameter 1.0 cm), and clear surrounding fat planes—a critical finding that was easily overlooked. Gynecologic pathology review revealed signet-ring cell infiltration of the cervix and endometrium with an enteric immunophenotype (CK20+++, SATB-2+++, CDX-2+++, CK7-, PAX-8-, ER-, PR-, Ki-67 85%), strongly suggesting gastrointestinal origin. Esophagogastroduodenoscopy and colonoscopy showed no lesions. The combination of uterine involvement with enteric immunophenotype and imaging suggesting appendiceal abnormality prompted surgical exploration (**Figure 1**).

Intraoperative findings (July 6, 2021) confirmed the diagnosis: markedly enlarged appendix adherent to its mesentery, with solid, yellow-white cut surface; uterus 16-week size, firm and brittle; miliary nodules on bilateral ovarian infundibulopelvic ligament peritoneum, bladder peritoneal reflection, and ileal mesentery; right ovarian involvement with spared left ovary—consistent with classic PMP dissemination pattern.

Final pathologic examination confirmed appendiceal signet-ring cell carcinoma with metastatic spread. According to the AJCC 8th edition staging system for appendiceal carcinoma, the tumor was classified as Stage IVC (pT4aNxM1). The primary tumor demonstrated transmural infiltration with serosal surface involvement without perforation (pT4a). Regional lymph nodes were not systematically dissected or evaluated (pNx), as the surgical procedure did not include right hemicolectomy with formal lymphadenectomy. Distant metastasis to the uterus, bilateral ovaries, omentum, and ileal mesentery was confirmed (pM1). The peritoneal cancer index (PCI) was retrospectively estimated as 16 based on detailed operative descriptions and photographic documentation, indicating moderate peritoneal tumor burden. Completeness of cytoreduction (CC) score was CC-0, with no visible residual disease following surgery.

### 2.5 Therapeutic intervention

Surgery: On July 6, 2021, the patient underwent open CRS, including subradical hysterectomy, bilateral salpingo-oophorectomy, appendectomy, partial omentectomy, lysis of adhesions, and ureterolysis. Operative time was 6.5 hours, with 1000 mL blood loss, transfusion of 3 units packed red cells and 600 mL fresh frozen plasma. Intraoperative peritoneal lavage with fluorouracil was performed.

Adjuvant Chemotherapy: From August 2021 to April 2022, the patient received 8 cycles of 5-fluorouracil (5-FU) (1000 mg, days 1-5) plus cisplatin (90 mg intraperitoneal, day 1). Grade 2 myelosuppression occurred during cycle 2; rash developed during cycle 8.

Post-Recurrence Treatment: First recurrence occurred in March 2024, treated with 6 cycles of 5-FU (1000 mg) plus carboplatin (450 mg) plus bevacizumab (500 mg) from March to September 2024, with good tolerance.

### 2.6 Follow-up and outcomes

The patient achieved 32 months of progression-free survival after initial surgery. Following recurrence and second-line chemotherapy, as of January 2026 (last follow-up), overall survival exceeded 52 months with good performance status and normalized tumor markers. No severe complications occurred within 30 days postoperatively.

## 3 Discussion

### 3.1 Diagnostic pitfalls: when the uterus becomes the “scapegoat”

This patient presented with vaginal bleeding and diffuse uterine enlargement, highly suggestive of primary uterine malignancy preoperatively. However, three critical clues indicated metastatic disease.

First, the imaging “blind spot.” CT showed appendiceal wall thickening with enhancement (transverse diameter 1.0 cm), which was overshadowed by prominent uterine findings. Normal appendiceal diameter is <6 mm; >10 mm with wall thickening suggests neoplastic infiltration rather than inflammation (19). Approximately 60% of appendiceal adenocarcinomas are misdiagnosed as appendicitis or ovarian tumors on preoperative imaging (20). For patients with “uterine enlargement with ascites,” routine appendiceal evaluation is essential.

Second, the “enteric fingerprint” in pathology. Preoperative curettage showed CK20+++/CK7-/PAX-8- immunophenotype. Primary uterine SRCC typically retains CK7 and/or PAX-8 expression (18, 21), while this case’s “completely negative” results (CK7-, PAX-8-, ER-, PR-) strongly pointed to metastasis. SATB-2, a specific nuclear transcription factor for the right colon and appendix, showed strong positivity (+++), further localizing the source to the cecal region (22).

Third, the “laterality” of dissemination. Right ovarian involvement with spared left ovary represents the hallmark of classic PMP dissemination. Appendiceal mucinous carcinoma follows a “right-sided preference” pattern due to anatomical continuity between the right paracolic gutter and right ovary, while the left side is blocked by the sigmoid colon (15). Bradley et al. (23) found 78% of ovarian involvement was right-sided or right-predominant in appendiceal mucinous tumors. For colorectal primaries, bilateral involvement is more common; for gastric cancer, symmetric bilateral involvement occurs as Krukenberg tumors (>80% bilateral) (24). This “right-heavy, left-spared” pattern became critical evidence for appendiceal origin.

### 3.2 Molecular pathogenesis of signet-ring cell carcinoma

While low-grade appendiceal mucinous neoplasms (LAMN) and well-differentiated mucinous adenocarcinomas characteristically harbor KRAS and GNAS mutations, appendiceal SRCC demonstrates a distinct molecular pathogenesis driven by genetic alterations promoting epithelial-mesenchymal transition (EMT) and loss of cellular adhesion (16). Recent genomic studies have revealed that SRCC of the appendix exhibits high-frequency TP53 mutations (60–80% of cases), chromosomal instability, and copy number alterations affecting cell adhesion molecules, contrasting sharply with the relatively stable genomes of LAMN (12, 14). These molecular features underlie the characteristic morphologic phenotype: loss of E-cadherin (CDH1) expression enables discohesive growth, while MUC2 and MUC5AC overproduction creates the abundant intracytoplasmic mucin vacuoles displacing nuclei to the periphery (25).

The pathogenesis of SRCC involves dysregulation of critical signaling pathways governing cellular differentiation and motility. Transforming growth factor-β (TGF-β) pathway activation plays a central role in promoting EMT, with downstream upregulation of ZEB1, SNAIL, and SLUG transcription factors suppressing E-cadherin expression and conferring invasive capacity (26). Concurrently, Wnt/β-catenin signaling alterations contribute to nuclear accumulation of β-catenin, driving proliferation and dedifferentiation toward the signet-ring cell phenotype (27) Whole-exome sequencing analyses have identified recurrent mutations in genes regulating the actin cytoskeleton (such as RHOA and RAC1), explaining the distinctive infiltrative growth pattern and propensity for peritoneal dissemination observed in SRCC (28).

Importantly, the molecular profile of appendiceal SRCC more closely resembles gastric SRCC than colorectal or appendiceal mucinous adenocarcinoma. Comparative genomic hybridization studies demonstrate shared amplification of chromosome 8q (containing the MYC oncogene) and 17q (containing ERBB2), alongside frequent CDH1 promoter hypermethylation or mutational inactivation (29, 30). This molecular convergence suggests that appendiceal SRCC may represent a distinct entity warranting classification separate from conventional appendiceal adenocarcinomas, potentially explaining its aggressive clinical behavior and poor response to standard colorectal chemotherapy regimens (31).

From a developmental perspective, two hypotheses exist regarding the histogenesis of appendiceal SRCC. The “*de novo*” hypothesis proposes direct malignant transformation from appendiceal crypt stem cells, bypassing the adenoma-carcinoma sequence characteristic of mucinous tumors (32). Alternatively, the “progression” model suggests high-grade transformation from pre-existing HAMN or poorly differentiated adenocarcinoma through acquisition of additional genetic hits (particularly TP53 loss and CDH1 inactivation) (33). Supporting the latter, occasional cases demonstrate mixed histology with both mucinous and signet-ring cell components, and molecular analyses reveal shared KRAS mutations between LAMN and adjacent SRCC in such instances (34).

The peritoneal dissemination pattern of SRCC reflects specific molecular adaptations. Overexpression of matrix metalloproteinases (MMP-2, MMP-9) facilitates basement membrane degradation, while upregulation of chemokine receptor CXCR4 promotes directed migration toward CXCL12-expressing mesothelial cells lining the peritoneal cavity (35, 36). Additionally, SRCC cells exhibit enhanced anoikis resistance through PI3K/AKT pathway activation, enabling survival as free-floating cells within the mucinous ascites characteristic of pseudomyxoma peritonei (37). These biological properties collectively explain the rapid peritoneal spread and difficulty in achieving complete cytoreduction in SRCC compared to well-differentiated mucinous tumors.

Therapeutically, the molecular landscape of SRCC presents both challenges and emerging opportunities. The frequent absence of actionable driver mutations (such as EGFR or BRAF alterations targetable in colorectal cancer) limits precision oncology approaches (38). However, the high mutational burden and chromosomal instability observed in some SRCC cases may confer susceptibility to immune checkpoint inhibitors, particularly in mismatch repair-deficient (dMMR) or microsatellite instability-high (MSI-H) subtypes, which occur in approximately 5–10% of appendiceal adenocarcinomas (39, 40). Ongoing clinical trials are evaluating programmed cell death protein 1 (PD-1) inhibitors in this molecular subset, with preliminary case reports suggesting durable responses (41).

### 3.3 Primary site localization: appendix vs. colorectum

**Figure 2:**
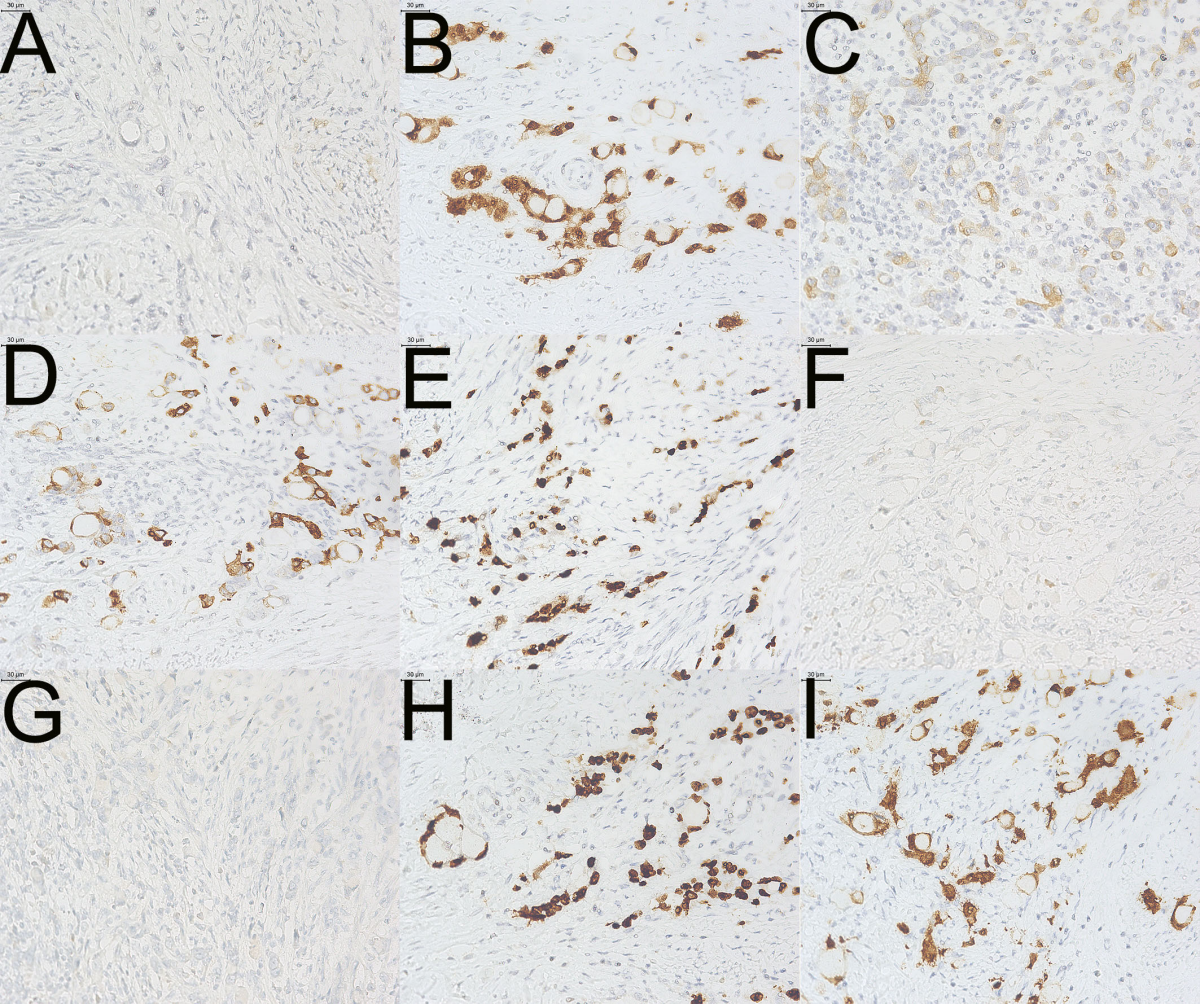
Immunohistochemical profile (200× magnification). **(A)** CA19-9: Negative staining in tumor cells. infiltration by signet-ring cell carcinoma. **(B)** CDX2: Positive nuclear and cytoplasmic staining (++). **(C)** CK7: Negative staining in tumor cells. **(D)** CK20: Strong positive cytoplasmic staining (+++). **(E)** Ki-67: High proliferation index (~85%). **(F)** PAX-2: Negative nuclear staining. **(G)** PAX-8: Negative nuclear staining. **(H)** SATB-2: Strong positive nuclear staining (+++). **(I)** Villin: Positive cytoplasmic and membranous staining.

This case was ultimately diagnosed as appendiceal SRCC (**Figure 2**), but faced differential diagnostic challenges with colorectal and gastric primaries both preoperatively and intraoperatively. This distinction affects not only pathological terminology precision but also directly impacts surgical extent (right hemicolectomy vs. simple appendectomy) and chemotherapy regimen selection.

#### 3.3.1 Immunohistochemistry: combined value of SATB-2 and CDX-2

Traditionally, CK20+/CK7- has limited discriminatory value between appendix and colorectum. SATB-2, a specific nuclear transcription factor for the right colon and appendix, has become key to differential diagnosis (42). It is positive in >90% of right-sided colonic and appendiceal adenocarcinomas, while virtually absent in gastric, pancreatic, biliary, and gynecologic tumors (43). This case’s dual strong positivity for SATB-2+++ and CDX-2++ strongly pointed to cecal region origin.

Subtle differences exist in staining patterns: CDX-2, an early marker of intestinal differentiation, often shows diminished expression in SRCC due to cellular dedifferentiation (only ++ in this case); while SATB-2, maintaining terminal differentiation, is better retained in signet-ring cell components (44). This “weak CDX-2/strong SATB-2” pattern is characteristic of appendiceal SRCC (16).

#### 3.3.2 Gross morphology and growth pattern

Intraoperative findings showed a solid, yellow-white, rigid appendix with luminal occlusion, distinct from typical mucinous cysts with “cystic dilation, gelatinous content.” This corresponds to infiltrative SRCC, rather than high-grade progression of low-grade appendiceal mucinous neoplasm (LAMN) (45). Appendiceal SRCC comprises <5% of appendiceal adenocarcinomas but shows the strongest invasiveness. Colorectal SRCC (1% of colorectal cancers) typically manifests as annular stricture or mass, rather than the tubular structure with diffuse sclerosis seen here (46).

#### 3.3.3 Molecular pathology: limitations

Appendiceal and colorectal SRCC share KRAS mutations (~40-60%), but appendiceal origin more frequently harbors GNAS mutations (~20%), associated with mucin secretion and peritoneal dissemination (47). This case’s genetic testing suggested “90% probability of esophageal or gastric origin,” contradicting the pathological diagnosis—reflecting limitations of commercial platforms: training databases are dominated by common tumors, with inadequate representation of rare tumors like appendiceal carcinoma (48). Histopathology and immunophenotype remain the gold standard.

#### 3.3.4 Confirmation by dissemination pattern

Right ovarian involvement with spared left ovary conforms to the “right-sided preference” of classic PMP; colorectal SRCC typically shows bilateral symmetry or random distribution, more frequently with liver/lung metastases (34). This case’s diffuse myometrial infiltration and ileal mesenteric nodules were consistent with appendiceal location and lymphatic drainage (49).

In summary, this case was ultimately diagnosed as appendiceal SRCC through triple evidence chain of immunophenotype (SATB-2+++/CDX-2++/CK7-/PAX-8-), gross morphology (solid, rigid appendix), and dissemination pattern (right ovarian priority, ileal mesenteric involvement). This diagnostic process suggests that for diffuse pelvic SRCC, systematic differential thinking incorporating “anatomic location-immunophenotype-dissemination pattern” should be established, avoiding diagnostic frameworks limited to single organs.

### 3.4 Treatment strategy: paradigm shift from “gynecologic malignancy” to “peritoneal surface malignancy”

This patient’s 4-year course progressed through initial CRS, adjuvant chemotherapy, and post-recurrence therapy, reflecting a shift from “gynecologic malignancy” to “peritoneal surface malignancy” management.

#### 3.4.1 Initial surgery: value and limitations

The initial surgery (July 2021) achieved complete gross cytoreduction (CC-0) with total hysterectomy, bilateral adnexa, appendix, partial omentum, and peritoneal nodule resection. Intraoperative recognition of appendiceal abnormality and immediate extended resection was critical (50). Complete CRS (CC-0/CC-1) is an independent prognostic factor for appendiceal PMP, with 5-year survival of 50-70% versus <20% for incomplete cytoreduction (51).

The initial surgery achieved CC-0 cytoreduction with a retrospectively estimated PCI of 16, placing the patient in the moderate tumor burden category (PCI ≤20). For appendiceal SRCC with PMP, PCI >20 is associated with significantly worse prognosis (5-year survival <20% versus 50–70% for PCI ≤20). The combination of moderate PCI (16) and complete cytoreduction (CC-0) likely contributed to the favorable 32-month progression-free survival observed in this case.

Two regrets: first, no right hemicolectomy. Appendiceal SRCC lymphatic drainage follows the ileocolic artery to paraaortic nodes; simple appendectomy may have inadequate lymph node dissection (52). Second, no hyperthermic intraperitoneal chemotherapy (HIPEC). HIPEC was unavailable in 2021; postoperative fluorouracil peritoneal lavage without thermal effect potentially compromised elimination of free cancer cells (53). CRS+HIPEC demonstrates 5-year survival >80% for well-differentiated appendiceal mucinous carcinoma (54), though HIPEC’s value remains controversial for SRCC subtypes (17).

#### 3.4.2 Adjuvant chemotherapy

The 8-cycle 5-FU plus intraperitoneal cisplatin regimen (August 2021–April 2022) drew from gastric cancer peritoneal metastasis experience (55), based on platinum sensitivity of SRCC and intraperitoneal administration for increased local drug concentration.

Retrospectively, oxaliplatin-based FOLFOX or FOLFIRI regimens may better align with colorectal biological nature. Lack of oxaliplatin or irinotecan may have compromised micrometastasis control—perhaps contributing to 32-month recurrence. Absence of bevacizumab warrants reflection: anti-angiogenic therapy’s value is established in metastatic colorectal cancer, and appendiceal SRCC similarly shows high VEGF expression (56).

#### 3.4.3 Post-recurrence treatment

Recurrence (March 2024) showed oligometastatic pattern (vaginal cuff, sacral ligaments, mesentery without distant metastases), providing a window for intervention (57). The 5-FU plus carboplatin plus bevacizumab regimen represented: (1) carboplatin substitution reducing toxicity; (2) bevacizumab targeting angiogenic microenvironment; (3) intravenous delivery improving tolerance.

This validated the “chronic disease management” concept—long-term tumor control through sequential intervention (58). Appendiceal SRCC with PMP typically shows median survival of 12–24 months; this case exceeded 52 months, attributable to: (1) initial complete cytoreduction; (2) limited recurrence without widespread carcinomatosis; (3) anti-angiogenic therapy.

#### 3.4.4 Implications for future treatment

Principles for appendiceal SRCC: initial complete cytoreduction with right hemicolectomy when indicated; postoperative FOLFOX/FOLFIRI with early bevacizumab; post-recurrence assessment of secondary cytoreduction; PCI scoring and tumor marker monitoring; multidisciplinary collaboration (59, 60). MMR/MSI status testing may provide therapeutic options with immune checkpoint inhibitors (39).

### 3.5 Implications for clinical practice

This case provides multidimensional lessons for preventing misdiagnosis and mistreatment.

First, “uterus-appendix” linked screening in imaging. This case’s CT appendiceal abnormality was masked by prominent uterine findings—a common “blind spot.” Mandatory appendiceal evaluation is recommended for: (1) diffuse uterine enlargement with myometrial thickening; (2) pelvic effusion/ascites with mucinous density (CT 10–30 HU); (3) poorly visualized right adnexal structures (19). Imaging reports should mandate: appendiceal diameter, wall thickness, and surrounding fat planes. MRI DWI can help—appendiceal SRCC shows high signal due to dense cellularity (61). Preoperative CA19–9 and CEA should be routine; elevated CA19-9 (>100 U/mL) suggests peritoneal dissemination risk, while CEA correlates with tumor burden (62).

Second, optimize pathological workflows. This case’s initial “endometrial simple hyperplasia” report delayed diagnosis by >2 months due to failure to recognize signet-ring cells and omit immunohistochemistry. “Uterus+gastrointestinal tract” bidirectional screening is recommended: CK7/CK20/PAX-8 triple staining for any atypical cells; if CK20+/CK7-/PAX-8-, immediate gastrointestinal endoscopy and consultation (22). Regional pathology consultation centers are critical for rare tumor diagnosis.

Third, intraoperative “three-step rule”: (1) Exploration—systematic inspection of appendix, cecum, stomach, colorectum, and diaphragm, recording PCI; (2) Biopsy—frozen pathology clarifying histologic type; (3) Planning—for signet-ring cell, pursue CC-0/CC-1 cytoreduction with right hemicolectomy when indicated; for low-grade mucinous, conservative management (63). PCI is a core prognostic indicator (5-year survival 60% vs. 20% for ≤20 vs. >20) (64).

Fourth, “biomarker+imaging” recurrence monitoring. Postoperative CEA, CA19-9, and CA-125 every 3 months is recommended; two consecutive increases >20% suggests peritoneal micrometastasis even without imaging findings (65). This case’s September 2023 examination found right sacral ligament thickening, but PET-CT confirmation was delayed 6 months—laparoscopic second-look might have enabled earlier intervention.

Fifth, institutionalize multidisciplinary team (MDT) model. This case’s success relied on “passively triggered” rather than “actively planned” collaboration. Appendiceal peritoneal malignancies should be routine MDT diseases with fixed multidisciplinary conferences (66). MDT values: preoperative unified diagnosis, individualized surgical planning, adjuvant therapy determination, and shared recurrence information.

Sixth, patient education and psychological support. Case manager introduction at diagnosis assists patients in understanding disease nature, treatment decisions, and financial assistance, with psychological screening during follow-up (67).

### 3.6 Limitations and future directions

Although this case report strives for comprehensiveness, several limitations remain, reflecting inherent constraints of single-center retrospective studies and highlighting research gaps in this rare disease field.

#### 3.6.1 Major limitations of this case

First, lack of systematic molecular assessment. Genetic testing reported only “90% probability of esophageal or gastric origin” without specific mutation spectrum (KRAS, NRAS, BRAF, GNAS, TP53). Low-grade appendiceal mucinous neoplasms (LAMN) often harbors GNAS/KRAS mutations, while high-grade SRCC shows TP53 mutations and chromosomal instability (45). These features guide targeted therapy—BRAF V600E for BRAF inhibitors, MSI-H/dMMR (mismatch repair-deficient) for immune checkpoint inhibitors (40). This case lacked MMR/MSI testing.

Second, retrospective PCI estimation. Although we have estimated PCI as 16 based on detailed operative records and specimen photography, prospective systematic scoring using the 13-region PCI classification was not performed at the time of surgery (68). This retrospective approach may introduce measurement inaccuracy and prevents reliable comparison with prospective studies employing standardized PCI documentation. Future cases should incorporate real-time PCI scoring by the surgical team.

Third, no secondary cytoreduction at recurrence. March 2024 recurrence showed oligometastatic disease (vaginal cuff, sacral ligaments, mesentery) without distant metastases, theoretically amenable to surgery. Secondary cytoreduction achieving CC-0/CC-1 extends median survival to 36–48 months versus 12–18 months for chemotherapy alone (69).

Fourth, lack of quality of life (QoL) data. Follow-up focused on tumor markers without standardized scales (EORTC QLQ-C30/QLQ-OV28) (70).

#### 3.6.2 Future research directions

First, establish multicenter registry for appendiceal SRCC. National registry with unified criteria, pathological standards, and treatment protocols is needed to provide evidence for guideline development (60).

Second, explore HIPEC value in SRCC. Current HIPEC evidence derives from low-grade tumors; SRCC is often considered relative contraindication. Prospective cohort studies comparing HIPEC versus non-HIPEC regimens are needed (71).

Third, optimize anti-angiogenic therapy. This case’s bevacizumab response aligns with ovarian cancer trials (72). Future exploration directions: (1) bevacizumab maintenance therapy duration (6 months vs. long-term); (2) anti-angiogenesis combined immune checkpoint inhibitor synergistic effects; (3) liquid biopsy VEGF level monitoring to guide therapy.

Fourth, artificial intelligence-assisted primary site localization. This case’s genetic testing “misjudgment” suggests traditional algorithms’ inadequate representation of rare tumors. Future multimodal AI model development is envisioned, integrating radiomics (appendiceal morphological features), pathomics (immunohistochemical staining patterns), and genomics (mutation characteristics), to improve primary site inference accuracy (73).

Fifth, routine incorporation of patient-reported outcomes (PRO). Clinical research and practice should place quality of life, symptom burden, treatment satisfaction and other PRO indicators alongside survival data, truly achieving patient-centered cancer care (74).

## 4 Conclusion

This article reports a case of appendiceal SRCC presenting with diffuse uterine involvement as the initial manifestation, diagnosed through surgery, pathology, and immunohistochemistry, with survival now exceeding 4 years. Key lessons include: (1) for patients with “uterine enlargement and ascites,” appendiceal morphology should be routinely evaluated, with immunohistochemistry CK20+/SATB-2+/CK7-/PAX-8- “enteric fingerprint” as the key to locking in metastatic source; (2) “laterality” of right ovarian priority dissemination is an important hallmark of classic PMP; (3) thorough CRS is the cornerstone of long-term survival, with post-recurrence anti-angiogenesis combined chemotherapy further extending survival; (4) genetic testing has limitations in rare tumor diagnosis, with histopathology remaining the gold standard; (5) multidisciplinary collaboration and standardized follow-up are critical. This case experience can inform diagnosis and management of similar cases.

## Data Availability

The original contributions presented in the study are included in the article/supplementary material. Further inquiries can be directed to the corresponding author.
